# Combined Damage Index to Detect Plastic Deformation in Metals Using Acoustic Emission and Nonlinear Ultrasonics

**DOI:** 10.3390/ma11112151

**Published:** 2018-11-01

**Authors:** Lu Zhang, Sepideh Karkouti Oskoe, Hongyu Li, Didem Ozevin

**Affiliations:** 1 Civil and Materials Engineering Department, University of Illinois at Chicago, Chicago, IL 60607, USA; zhang899@uic.edu (L.Z.); 3pde.karkouti@gmail.com (S.K.O.); dozevin@uic.edu (D.O.); 2 College of Civil Engineering and Architecture, Guilin University of Technology, Guilin 541004, China; 3 Collaborative Innovation Center for Exploration of Hidden Nonferrous Metal Deposits and Development of New Materials in Guangxi, Guilin University of Technology, Guilin 541004, China

**Keywords:** failure mechanism, acoustic emission, linear ultrasonic testing, nonlinear ultrasonic testing, combined NDT method

## Abstract

Understanding the amount of degradation using nondestructive evaluation (NDE) methods provides an effective way of determining the fitness to service and the residual life of structural components. Due to uncertainties introduced by the single NDE method, a combined damage index using multi-sensor data increases the reliability of damage assessment. In this paper, the outputs of three NDE methods including acoustic emission (AE), linear ultrasonics (LUT), and nonlinear ultrasonics (NLUT) are merged to identify the amount of plastic deformation in aluminum 1100. The sensitivities of individual and combined methods to microstructural changes are evaluated. The coupon samples are loaded up to different strain levels and then unloaded. AE data is recorded in real time and ultrasonic data is recorded from the unloaded samples. The major features combined in the damage index are cumulative AE absolute energy and nonlinear coefficient. The microstructural state is verified with microscopic analysis and hardness testing. The developed damage index can nondestructively assess the amount of plastic deformation with higher reliability.

## 1. Introduction

Aluminum is the second-most used metal due in part to its superior mechanical performance and versatility [1]. The applications of this metal have been especially widespread and common within the aerospace and automobile industries. However, the aluminum metal also has adversely inherent factors that could influence the mechanical performance of structural components, such as inner defects caused by the mechanical process or surface faults caused from external impact, possibly leading to irretrievable fractures. Meanwhile, the failure of the aluminum usually starts from the accumulation of microdefects due to complex loading [2]. Aluminum 1100 has higher aluminum contents than other aluminum alloys and the evolution of the damage mostly shows ductile behavior and involves plastic strain accumulation [3]. In the microscale, the grain boundary changes can be attributed as the main reason of failure. Without understanding of damage behavior and evolution, unexpected failures could happen. It is thus very important to find appropriate methods to detect the changes of microdefects within the material, while also quantifying the evolution of damage, from the time during its manufacture through its service life. Though microstructure assessment techniques (e.g., scanning electron microscopy (SEM), X-ray spectroscopy (EDS) etc.) are powerful and provide quantitative tools in failure analysis, the rigorous requirement of sample preparation, complex devices, destructive nature, and off-line investigation hinder in-situ implementation. Nondestructive testing (NDT) can provide a feasible and efficient option to evaluate the damage process, which usually involves a dynamic process. Acoustic emission (AE) is one of the NDE methods, which is widely used for damage detection in metals. It is a real-time NDE method and has the ability to sense the microstructure changes [4,5]. The method is based on detecting elastic waves released from active flaws. The method is typically considered qualitative in terms of damage size and severity. Aiming at the microstructure changes (grain boundary slipping, migration of dislocation, twinning, phase transformations) in the metallic materials, the AE method has many successful application examples [2,5,6,7,8,9,10]. Ultrasonic testing (UT) is an active NDE method and is applied mostly as periodic assessment [11]. It is a post-test NDE method, and provides quantitative information about damage (e.g., thickness loss, crack size/location). UT is classified as linear UT (LUT) and nonlinear (NLUT) methods. LUT as a conventional method is based on using a single frequency to detect the defects, which can cause amplitude and phase change in signal due to scattering. The information obtained from LUT has limited sensitivity compared with NLUT [12]. The concept of NLUT is to identify the elastic wave distortion due to material nonlinearity (e.g., microcrack, plastic deformation, porosities). When a single-frequency wave propagates in a material, higher-frequency components (higher harmonics) are generated by interaction between the single frequency mode and microstructural changes [13]. For testing aspect, the receiver transducer is usually tuned to second or third harmonic frequency of transmitting transducer to sense the nonlinearity.

The integration of multiple sensing techniques increases the reliability of NDE. Aggelis et al. used both AE and UT to identify damage in composite materials [14]. Shah and Ribakov measured the nonlinearity using UT and further used AE to evaluate the distributed damage in concrete [15]. The correlation between the AE and UT methods is investigated to assess damage. Ohtsu et al. quantified the defects in concrete using both methods [16]. The AE method is used to localize cracks and classify crack types based on the estimation of defects using UT. Turner et al. determined the effect of distance from the near-contact ultrasonic tool using acoustic emission, and then the proper standoff position could be chosen accordingly [17]. However, there is no study in the literature on combining AE and UT for assessing the microdamage evolution in ductile materials. 

In this paper, the outputs of AE, LUT, and NLUT are merged to identify the amount of plastic deformation in aluminum 1100. The sensitivities of individual and combined methods to microstructural changes are evaluated. The coupon samples are loaded up to different strain levels and then unloaded. AE data is recorded in real time and ultrasonic data is recorded from the unloaded samples. The major features studied to define the damage index are cumulative AE absolute energy, linear attenuation coefficient, and nonlinear coefficient. The microstructural state is verified with microscopic analysis and hardness testing. The developed damage index can nondestructively assess the amount of plastic deformation. The outline of this paper is as follows. The material preparation and experimental procedure are described in Section 2. The methodology, results and discussion of NDT methods and the individual, combined damage index are presented in Section 3. The conclusions are summarized in Section 4.

## 2. Materials and Test Procedures

### 2.1. Description of Material and Samples

**Figure 1:**
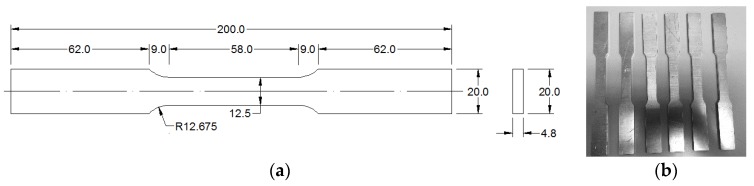
(**a**) The dimensions of tensile specimens (unit: mm) and (**b**) sample set.

Al 1100 used in this study has the composition of 99% Al and 1% Si + Fe [18]. All of the specimens were taken from the same plate with thickness of 4.8 mm. Coupon specimens were based on the ASTM E8 standard [19] with 50 mm gauge length, 12.5 mm width, and 4.8 mm thickness. The detailed dimensions of sample are shown as Figure 1a. A total of six samples were prepared and manufactured for the tensile tests, see in Figure 1b. The samples were numbered as S1 to S6. Before the tensile test, the stress relief procedure was conducted to eliminate the influence of internal (residual) stress due to machining. The samples were annealed at 300 °C for one hour in furnace, and subsequently air-cooled for 24 h.

### 2.2. Experimental Setup

**Figure 2:**
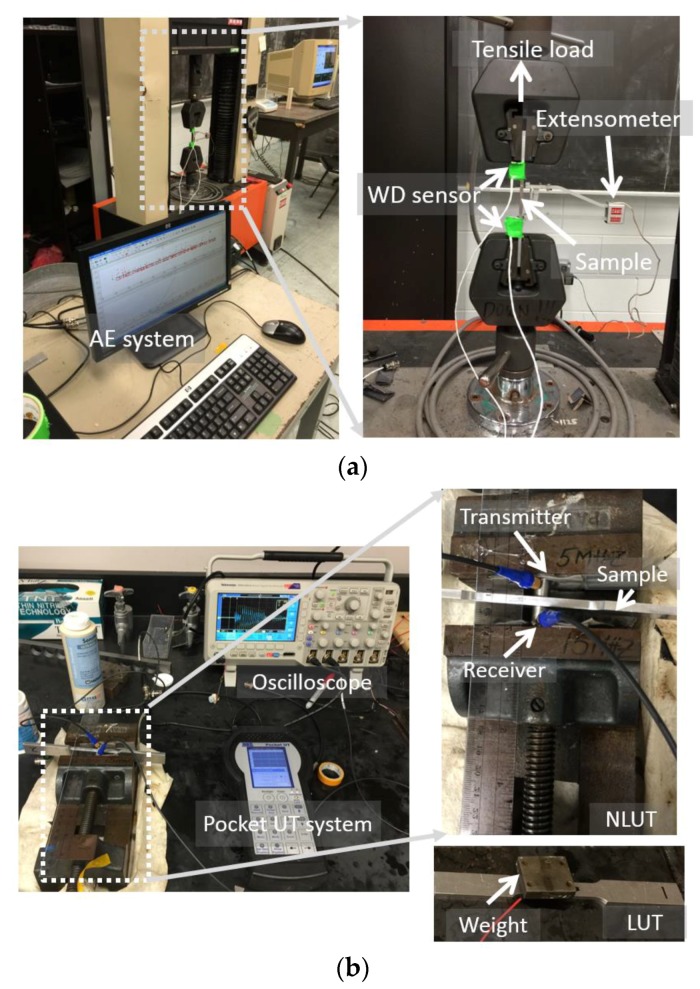
The experimental setups; (**a**) tensile test and acoustic emission (AE) setup and (**b**) ultrasonic testing (UT) measurements including linear ultrasonics (LUT) and nonlinear ultrasonics (NLUT).

In order to generate the different strain levels, each specimen was loaded up to certain strain level and then unloaded using Instron electromechanical load frame machine as shown in Figure 2a. Elongation was measured by an extensometer over the gauge length of 50 mm. The uniaxial loading was applied at the rate of 0.02 mm/s for S1. The loading rate was adjusted to 0.04 mm/s for S2 to S6 in order to increase the energy release of damage from ductile material and reduce the influence of grip friction. For S1, the sample was loaded up to failure; and strain–stress curve was used to determine yield strength, tensile strength, and the Young’s modulus. The exposed strain levels for S2 to S6 were 1%, 2%, 6%, 10%, and 14%, respectively.

The nondestructive testing consisted of online and offline methods: (i) the online monitoring setup includes recording AE and elongation continuously during the tensile test, see in Figure 2a, and elongation can be converted into strain data; (ii) the ultrasonic testing including linear ultrasonics (LUT) and nonlinear ultrasonics (NLUT) was implemented under no load as offline methods, see in Figure 2b. For AE testing, two piezoelectric sensors (WD sensor, manufactured by Mistras Group Inc., Princeton Junction, NJ, USA) were attached on both ends of gauge length. High vacuum grease was used as the couplant. The application frequency of WD sensor ranges from 20 kHz to 1 MHz. All of the sensors were connected to 40 dB gain pre-amplifiers. The AE data was continuously recorded using PCI-8 data acquisition board manufactured by Mistras Group Inc. The major data acquisition variables were the sampling frequency as 1 MHz, peak definition time as 200 µs, hit definition time as 800 µs, hit lockout time as 1200 µs, 40 dB as threshold, and the analog filter ranged from 20 kHz to 400 kHz.

The UT measurement was performed using pocket UT device manufactured by Mistras Group Inc. The setup is shown in Figure 2b. For the LUT, the pulse-echo mode was used, and longitudinal transducer with central frequency of 10 MHz was used. For NLUT, through transmission mode was used: the transmitted signal was generated by the transducer with frequency of 5 MHz, and the propagating wave was detected using the transducer with frequency of 10 MHz. A tone burst signal of ten cycles was used as the excitation signal and recorded using MSO2014 oscilloscope (100 MHz sampling frequency) manufactured by Tektronix (Beaverton, OR, USA). For both LUT and NLUT, light lubrication oil was used as couplant for attaching the transducers. As the method is applied after unloading, the results represent the accumulation of damage at the end of loading/unloading cycle. For each sample, the measurement was conducted near the middle section of gauge length and repeated three times with recoupling the transducers to check the repeatability and determine the recoupling error. In order to obtain consistent coupling force and reduce the operational error, constant weight and an adjustable clamp were used to press the transducer for LUT and NLUT, respectively, as shown in Figure 2b.

## 3. Results and Discussion

### 3.1. Characteristics of Mechanical Behaviors

#### 3.1.1. Tensile Testing

**Figure 3:**
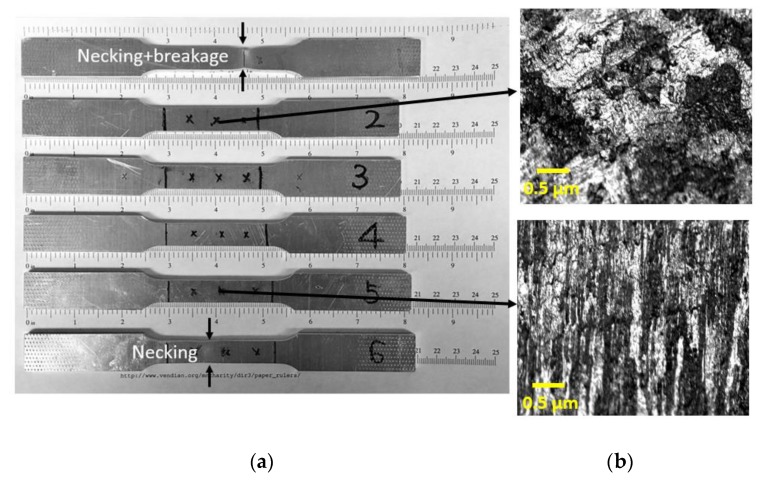
(**a**) The sample profiles after loading/unloading and (**b**) microscopic images.

**Figure 4:**
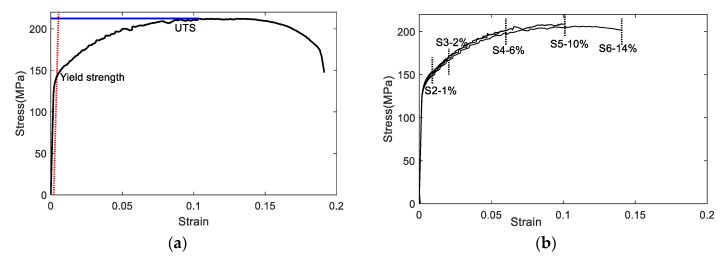
Strain–stress curves of (**a**) S1 and (**b**) S2 to S6.

The sample profiles after tensile tests are shown in Figure 3. As mentioned above, the S1 was tested up to failure. The necking occurred before fracture, and the obvious necking was observed in S6 (14% strain); however, there was no necking in S5 (10% stain). It indicates that the necking starts in the strain level ranged from 10% to 14%. Thus, the UTS strain was adjusted as 12%. In addition, the yield strength, ultimate tensile strength, and modulus of elasticity were determined based on the results of S1. The material properties are summarized in Table 1. Figure 4 shows the stress–strain curve of each sample highlighting the stress–strain points that the samples were loaded/unloaded. Good repeatability in stress–strain behavior was observed. The microstructure analyses of two samples (S2 and S5) were conducted. The process follows the standard metallographic procedure [20]. The microscopic images were taken from the middle of the gauge. The microstructure features are shown in Figure 3. During the tensile process, the grain shows dynamic recrystallization behavior. The grain was elongated and reoriented due to increase of strain level. The plastic deformation is mainly attributed to the grain change in the tensile test; in addition, the acoustic nonlinearity is attributed to the grain morphology.

#### 3.1.2. Hardness Testing

**Figure 5:**
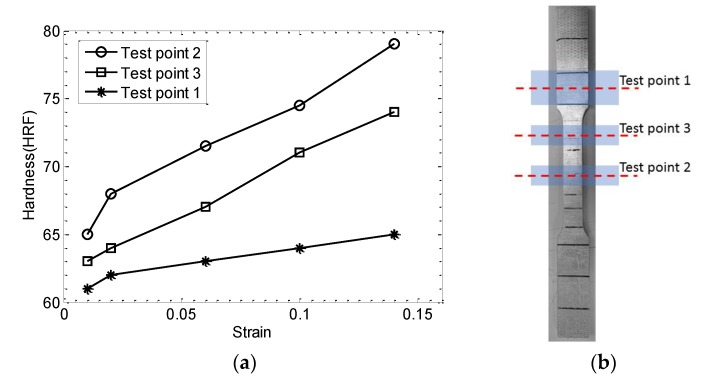
(**a**) The relationship between strain and hardness (HRF), (**b**) testing points.

Hardness is a measure of how resistant a material is to permanent shape change when a compressive force is applied [21], which depends on ductility, elastic stiffness, plasticity, strain, strength, toughness, viscoelasticity, and viscosity. It represents an arbitrary quantity used to provide a relative idea of material properties. Therefore, hardness can only offer a comparative idea of the material’s resistance to plastic deformation [22]. The Rockwell test was used to determine the hardness in this case; and three tested positions were selected in each sample. The tested points are shown in Figure 5b, and the corresponding hardness results are summarized in Table 2, and plotted in Figure 5. As expected, the hardness is correlated well with the plastic deformation. It is expected that the position of grips would not be exposed to plastic deformation. A slight change of hardness at grip point is observed while more significant increase in hardness is measured at gage length. Hardness values are combined with NDE results to give the combined damage index.

### 3.2. Real-Time Monitoring of Damage Evolution: Acoustic Emission (AE)

**Figure 6:**
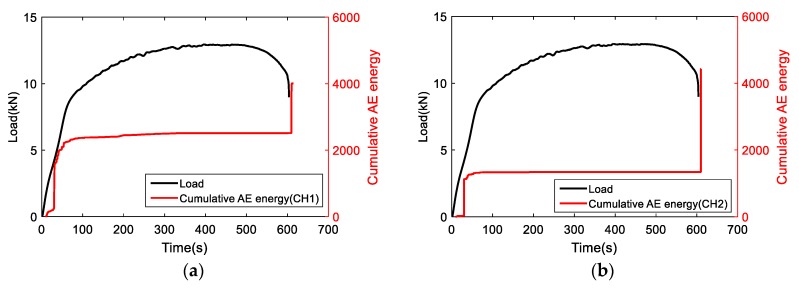
The correlation of load and cumulative AE energy for (**a**) CH1 and (**b**) CH2.

As discussed above, the evaluation of damage progress consisted of real-time and post-test assessment methods. Plastic deformation is the main AE source for the tensile loading of aluminum [23]. Firstly, AE signals were synchronized with the stress–strain curve. Figure 6 shows the cumulative AE energies obtained from two AE sensors. The cumulative AE energy has similar behavior as mechanical response. At the beginning of loading, the specimen experienced elastic to plastic deformation with the AE energy accumulating quickly with the load increase. After 437.5 s associated with the UTS strain, the load starts to reduce due to necking. No significant AE was detected at strain hardening and necking. At the end of loading, a jump in AE energy occurred coinciding with the fracture. The process of AE activity is then divided into three stages: initiation (start–67.9 s), stable (67.9 s–600 s), and failure (600 s–end).

**Figure 7:**
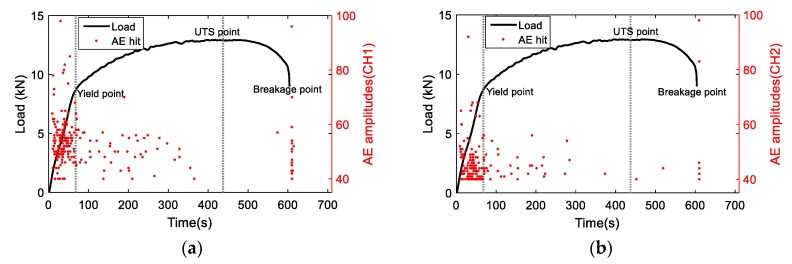
The AE hit distribution versus loading history (**a**) CH1 and (**b**) CH2.

The distribution of AE hits with the loading information is shown in Figure 7. Similar trends as for AE energy are observed. The majority of AE hits are detected in the initial and failure stages. The AE hits at the initial stage may be attributed to dislocation slip or fiction from grip area. The AE hits at the failure stage are due to fracture. As two channels have similar responses, the AE signals obtained from CH1 are further analyzed.

**Figure 8:**
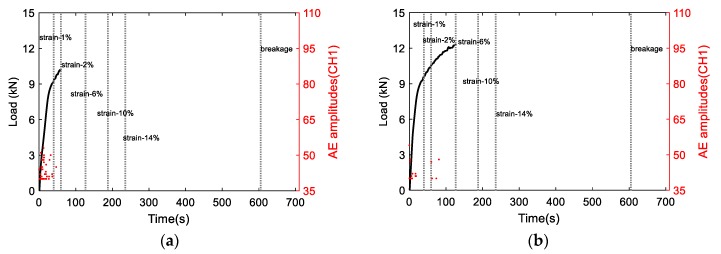

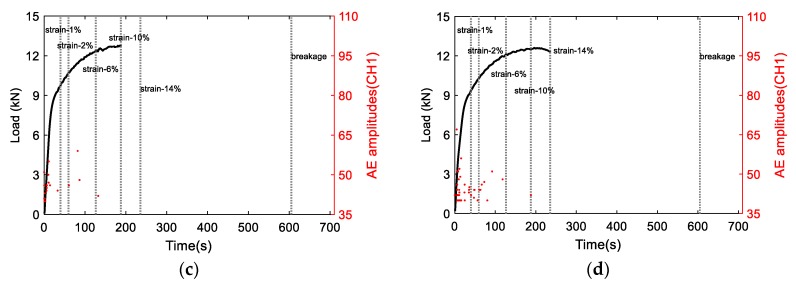
The AE amplitudes with respect to load curve for (**a**) S3, (**b**) S4, (**c**) S5, and (**d**) S6.

**Figure 9:**
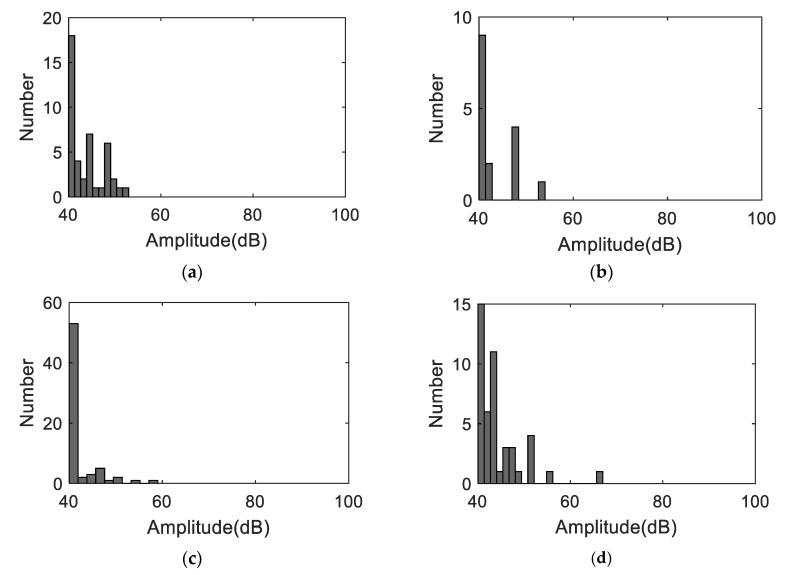
The AE amplitude histograms (**a**) S3, (**b**) S4, (**c**) S5, and (**d**) S6.

**Figure 10:**
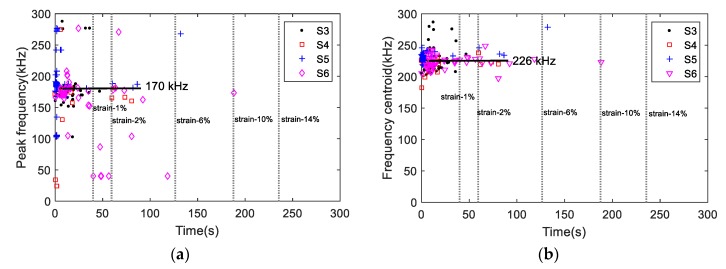

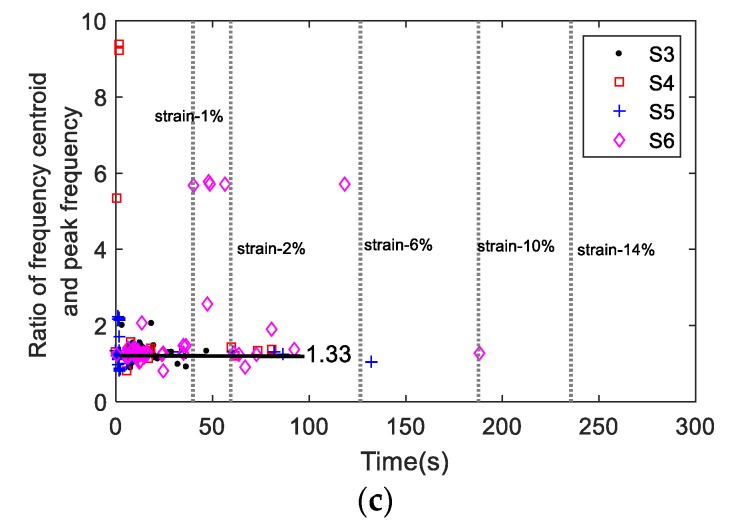
The frequency-related AE features (**a**) peak frequency, (**b**) frequency centroid, and (**c**) the ratio of frequency centroid and peak frequency.

Figure 8 shows the AE amplitudes with respect to loading for S3 to S6, and Figure 9 shows the AE amplitude histograms of S3 to S6. The AE data was not recorded properly from S2 due to improper sensor attachment; therefore, the AE data from S2 was not used. The AE amplitudes are less than 65 dB as expected due to the ductile behavior of Al 1100. High amplitude signals are detected close to fracture similar to S1 as shown in Figure 7. However, the AE activity of S1 up to the yielding point is different than the AE activities of S3 to S6 due to different loading rate. The AE hits depend on the loading history and rate [24]. The frequency-related features are plotted in Figure 10. The peak frequency is obtained from the frequency spectrum of time history signal. The frequency centroid represents the centroid of frequency spectrum with respect to spectrum amplitude. Until the strain level of 6%, the AE hits from different samples show similar frequency content; herein, peak frequency and frequency centroid are near 170 kHz and 226 kHz, respectively. Typically, AE signals due to mechanical friction are expected to occur at lower frequencies [25]. The range depends on the resonant frequency bandwidth of AE sensor selected. The ratio of frequency centroid to peak frequency is near 1.3 (Figure 10c), which indicates that the frequency spectrum has higher frequency components in addition to peak frequency. Therefore, the AE activities within strain level of 6% can be attributed to the same AE source mechanism, which is caused by the initial accumulation of microstructure change, such as dislocation slip or grain change. The microscopic image can provide the evident, see in Figure 3. The grain profile with low load is an equiaxed structure, with the increase of load, the grain is elongated. Though there is no direct evidence which indicates the AE is caused by the grain change, many studies in the literature already demonstrate that the metallic grain change (e.g., the grain angle change [26] and grain size change [23]) generates AE activities. In the end of the test, high AE energy released caused by rupture, see Figure 7.

**Figure 11:**
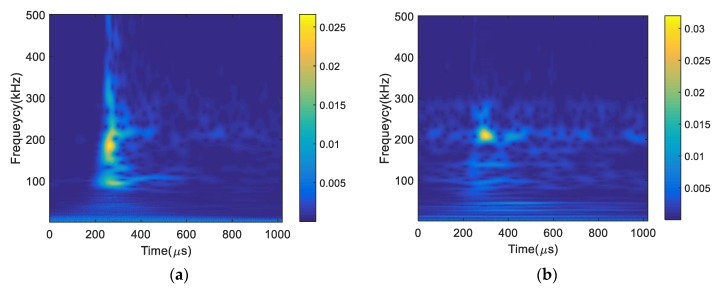
Spectrograms of AE signals obtained at different load levels (**a**) strain < 6% and (**b**) breakage.

In order to further characterize AE signals from different strain levels, the spectrograms of AE signals are obtained using complex wavelet transform, shown in Figure 11. The AE signal obtained at strain level below 6% has the peak frequency near 200 kHz, similar to fracture energy, in addition to low frequency components. This may be due to the contribution of friction as well as dislocation movement in a single AE signal. Though the AE sources could not be identified directly in this study, similar findings have been obtained in the literature [27,28]. The spectrogram of the AE signal obtained near fracture has high concentration near 200 kHz. It is important to note that the frequency bands highly depend on the AE sensor selected in addition to source mechanism. In general, high frequency components are attributed to the internal microstructure change, such as grain elongation, dislocation, and fracture [25].

**Figure 12:**
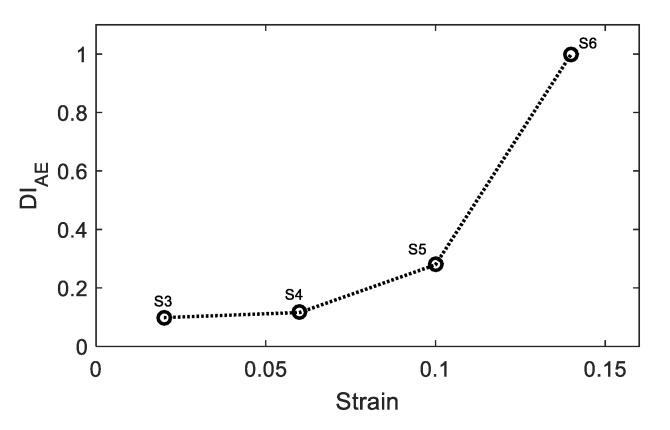
The relationship between AE damage index and strain.

It is important to define a damage index (DI) correlated to the exposed plastic deformation. DI is defined based on the average cumulative absolute energy at the end of each loading normalized to the average cumulative absolute energy obtained from the fractured sample (reference sample), and calculated as Equation (1):

(1) DIAE=∑i=1nAbs Engin∑i=1mAbs Engim

where the numerator represents the cumulative AE absolute energy, and n represents number of hits recorded up to particular strain level. The denominator represents the cumulative AE absolute energy and m represents number of hits recorded up to fracture. DI results are shown in Figure 12. There is good correlation between the AE-based damage index and the level of plastic deformation.

### 3.3. Post-Test Evaluation of Damage Evolution: Ultrasonics

The correlations between LUT attenuation and NLUT acoustic coefficient with different plastic strains are sought. The major LUT outputs related to the damage state are attenuation properties and wave velocity. The nonlinear harmonic generation technique is used to measure the acoustic nonlinearity parameters (ANP). When a sinusoidal ultrasonic wave propagates through a nonlinear medium, higher harmonics are produced due to heterogeneity. The acoustic nonlinearity parameter (ANP) for the third harmonic generation is defined to characterize the nonlinearity as [29]:

(2) $$\gamma =\frac{6A_{3}}{A_{1}^{3}{\mathrm{xk}}^{3}}$$

where, $A_{1}$ is the amplitude of the fundamental harmonic wave; $A_{3}$ is the amplitude of the third harmonic wave; x is the propagation distance, in this case (x equals the thickness of the specimen), and k is the wave number which is constant for each sample. For longitudinal waves with a fixed frequency, the ANP (γ) is only proportional to $\frac{A_{3}}{A_{1}^{3}x}$. Therefore, in this measurement, a simplified relative ANP is defined as damage index for NLUT as

(3) $${\mathrm{DI}}_{\mathrm{NLUT}}=\frac{A_{3}}{A_{1}^{3}x}$$

**Figure 13:**
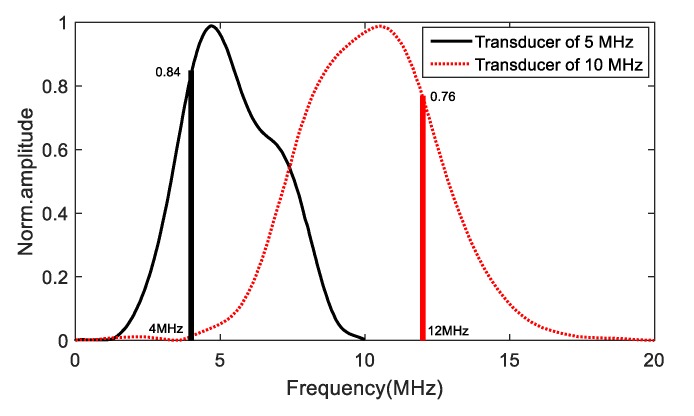
The sensitivity curves of UT transducers provided by manufacturer (Olympus Inc., Waltham, MA, USA).

**Figure 14:**
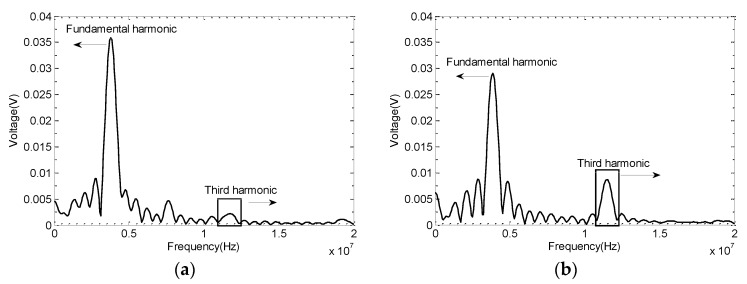
The fundamental and third harmonics of the received ultrasonic signals from (**a**) S2 with 1% strain and (**b**) S6 with 14% strain.

The 5 MHz ultrasonic transducer was used as transmitter, and the frequency of the receiver was 10 MHz. In order to generate the third harmonic with the current transducer configuration, an excitation signal of 4 MHz was used, and the received signals show good resolution at the third harmonic frequency using the transducer with 10 MHz frequency (Figure 13 and Figure 14). As shown in Figure 13, though the amplitude corresponding to 4 MHz and 12 MHz is smaller than the resonant frequency, the sensitivity is sufficient to detect the third harmonics with the current transducer configuration.

Figure 14a,b shows the frequency spectra of UT signals obtained from S2 and S6. The fundamental ($A_{1}$) and third harmonic ($A_{3}$) frequencies are highlighted on the plots. An increase in the third harmonic frequency with the increase of plastic deformation is clearly observed. Usually, the microstructure change due to the plasticity is reflected by the second harmonic’s generation [30]. The third harmonic usually has higher sensitivity for characterizing the grain boundaries due to weak nonlinearity [31].

**Figure 15:**
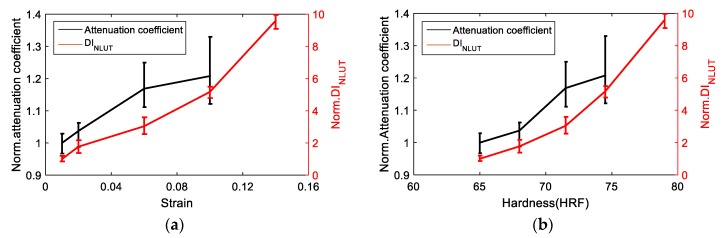
Correlation of UT outputs with, (**a**) plastic strain and (**b**) hardness.

Figure 15a,b shows the correlation of strain level and hardness with ultrasonic attenuation coefficient and nonlinearity, respectively, normalized to pristine sample. LUT attenuation and NLUT acoustic nonlinearity coefficients show similar behaviors with the increase of strain. As shown in the metallographic images of S2 (1% strain) and S5 (10% strain) in Figure 3, the grain distribution changes with plastic deformation, which influences the ultrasonic properties. With increasing strain level, the grain becomes narrowed and elongated. Those microstructural changes are correlated with the ultrasonic properties. However, the errors in linear attenuation coefficient are higher while its sensitivity to plastic deformation is lower than the nonlinearity coefficient, as expected. Therefore, the damage index obtained from NLUT is used in combination with the damage index obtained from AE.

### 3.4. The Results of Combined AE, UT, and Strains Level

**Figure 16:**
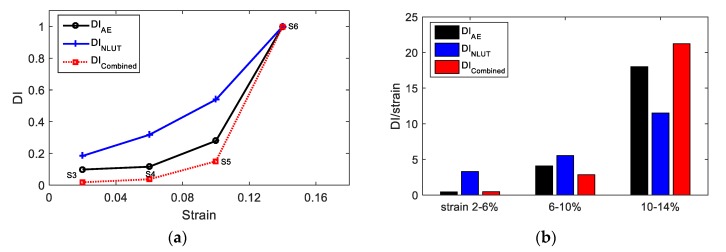
(**a**) The correlation of strain level, AE damage index, NLUT damage index, and combined AE and UT damage index; (**b**) the sensitivity of DI.

AE and UT parameters can represent the damage level individually or concurrently. The correlations of DI_AE_ and DI_NLUT_ are shown in Figure 16a. Both methods indicate good relations with the increase of plastic deformation. However, they have measurement uncertainties and error sources (e.g., background noise for AE and the influence of secondary sources to UT measurement such as surface roughness). The combined DI can effectively increase the reliability and accuracy of the detection and is defined as Equation (4):

(4) $${\mathrm{DI}}_{\mathrm{combined}}=\overset{n}{\underset{k=1}{\prod }}{\mathrm{DI}}_{k}$$

The combined damage index of AE and NLUT measurement is shown in Figure 16a. As shown in this figure, with the increase of plasticity in the material, the DI increases. The rate of material degradation is higher when the material approach to failure. The sensitivity is defined as the ratio of DI and strain, see in Figure 16b. With the increase of damage in the material, the sensitivity of DI increases for three measurements. For the initial stage of testing (0–10%), DI_NLUT_ has the highest sensitivity because the NLUT is more sensitive to the microstructure change in the initial stage. DI_AE_ and DI_combined_ are more sensitive when the material approaches failure.

## 4. Conclusions

In this paper, the passive and active NDE methods were utilized concurrently to assess the plastic deformation in aluminum 1100 such that each method could be combined for more reliable damage assessment. The damage index equations for individual and combined NDE methods were defined. For the AE testing as a real-time and in-situ NDE method, three major regions with different AE activities were determined: elastic-plastic region, strain hardening-necking region, and failure region. Most activities were detected in the elastic-plastic region. Due to ductile behavior of Al 1100, no significant AE were detected at the strain hardening-necking region. The AE-based damage index was defined as the cumulative value of AE absolute energy at the end of loading. The sensitivities of linear and nonlinear ultrasonics to plastic deformation were studied. While both methods indicated an increase in damage index, linear ultrasonics has higher variation in repeated measurements. The damage index of nonlinear UT was defined using the fundamental and third harmonic frequencies. In general, both AE and nonlinear UT methods have good relations with the increase of plastic deformation. However, the combined damage index reduces the influence of error sources introduced by individual NDE method. The concept shows the potential of combined AE and UT methods in the damage assessment of large-scale structures. The AE method as a real-time method provides qualitative information and can pinpoint the location of major damage, which can be validated further and quantified with the in-situ nonlinear UT method.

## Figures and Tables

**Table 1:** Mechanical properties obtained from the strain–stress curve of S1.

Properties	Young’s Modulus E (GPa)	Poisson Ratio ν	ρ (kg/m^3^)	σ_ys_ (MPa)	Strain (Yield)	UTS (MPa)	UTS Strain	Elongation (%)
Value	64	0.33	2710	142.7	0.0042	212.5	0.12	19

**Table 2:** Hardness results (HRF).

Sample	Test Point 1	Test Point 2	Test Point 3
S2 (1%) *	61.0	65.0	63.0
S3 (2%) *	62.0	68.0	64.0
S4 (6%) *	63.0	71.5	67.0
S5 (10%) *	64.0	74.5	71.0
S6 (14%) *	65.0	79.0	74.0

* strain level that sample was exposed to.

## Nomenclature

NDE	Nondestructive evaluation
AE	Acoustic emission
LUT	Linear ultrasonics
NLUT	Nonlinear ultrasonics
UT	Ultrasonic testing
SEM	Scanning electron microscopy
UTS	Ultimate tensile strength
ANP(γ)	Acoustic nonlinearity parameters
DI	Damage index
µs	Microsecond
*E*	Young’s modulus
ν	Poisson ratio
ρ	Density
*Abs Eng.*	AE absolute energy
